# Identification of stress-responsive genes in *Ammopiptanthus mongolicus* using ESTs generated from cold- and drought-stressed seedlings

**DOI:** 10.1186/1471-2229-13-88

**Published:** 2013-06-05

**Authors:** Meiqin Liu, Jing Shi, Cunfu Lu

**Affiliations:** 1 Analysis and Testing Center, Beijing Forestry University, Beijing, 100083, China; 2 College of Biological Sciences and Biotechnology, Beijing Forestry University, Beijing, 100083, China

**Keywords:** *Ammopiptanthus mongolicus*, Cold tolerance, Differential expression, Drought tolerance, Expressed sequence tags, Stress-responsive genes

## Abstract

**Background:**

*Ammopiptanthus mongolicus* is the only evergreen broadleaf shrub in the northwest desert of China, which can survive long-term aridity and extremely cold environments. In order to understand the genetic mechanisms underlying stress tolerance and adaptation to unfavorable environments of woody plants, an EST approach was used to investigate expression patterns of *A. mongolicus* in response to abiotic stresses.

**Results:**

ESTs were generated from a cDNA library constructed from *A. mongolicus* seedlings subjected to cold and drought stresses. Analysis of 5,637 cDNA sequences led to the identification of 5,282 ESTs and 1,594 unigenes, which were denoted as the AmCDUnigene set. Of these, 70% of unigenes were annotated and classified into 12 functional categories according to Gene Ontology, and 30% of unigenes encoded unknown function proteins, suggesting some of them were novel or *A. mongolicus* specific genes. Using comparative analysis with the reported genes from other plants, 528 (33%) unigenes were identified as stress-responsive genes. The functional classification of the 528 genes showed that a majority of them are associated with scavenging reactive oxygen species, stress response, cellular transport, signal transduction and transcription. To further identify candidate abiotic stress-tolerance genes, the 528 stress-responsive genes were compared with reported abiotic stress genes in the Comparative Stress Genes Catalog of GCP. This comparative analysis identified 120 abiotic stress-responsive genes, and their expression in *A. mongolicus* seedlings under cold or drought stress were characterized by qRT-PCR. Significantly, 82 genes responded to cold and/or drought stress. These cold- and/or drought-inducible genes confirmed that the ROS network, signal transduction and osmolyte accumulation undergo transcriptional reorganization when exposed to cold or drought stress treatments. Additionally, among the 1,594 unigenes sequences, 155 simple sequence repeats (SSRs) were identified.

**Conclusion:**

This study represents a comprehensive analysis of cold and/or drought stress-responsive transcriptiome of *A. mongolicus*. The newly characterized genes and gene-derived markers from the AmCDUnigene set are valuable resources for a better understanding of the mechanisms that govern stress tolerance in *A. mongolicus* and other related species. Certain up-regulated genes characterizing these processes are potential targets for breeding for cold and/or drought tolerance of woody plants.

## Background

Environmental degradation, especially desertification, has become a serious problem due to the world’s population increases, the extensive agricultural practices and the effects of the global climate changes. The proliferation of woody plants in arid and high latitude deserts is essential for sustainable conservation and amelioration of environments. Genetic improvement of forest trees for tolerance to environmental stresses such as drought, high salinity, low temperature and heat is a promising approach for the future of environmental conservation. It is vital to know how plants respond to environmental stresses and which biochemical pathways and genes are involved in stress tolerance. To date, numerous physiological and biochemical traits have been observed, and hundreds of underlying genes have been characterized according to the recent developments in functional genomics of some herbaceous model plants such as *Arabidopsis*[1,2] and rice [3,4]. However, molecular mechanisms by which woody plants respond to abiotic stresses remain poorly understood despite their biological and practical importance.

The genus *Ammopiptanthus* (Leguminosae), endemic to the eastern desert of Central Asia, includes two species: *A. mongolicus* (Maxim.) Cheng f. and *A. nanus* (M. Pop.) Cheng f. [5]. *Ammopiptanthus* is the only genus of evergreen broadleaf shrub in the northwest desert of China and both species are dominant in the local vegetation, so this genus plays an important role in fixing moving sands and delaying further desertification. The biological property of *Ammopiptanthus* evergreen broadleaf has been viewed as an ancestral trait that identifies it as a Tertiary relict taxon [6]. The vegetation in northwest China was dominated by evergreen and/or deciduous broadleaf forests in the early Tertiary period according to the fossil evidence [7]. When subsequent changes made the climate colder and drier from the early Miocene (24–16 Ma) in Central Asia [8], the forests were gradually replaced by steppe and then by desert [9]. Today, their habitats are stony and/or sandy deserts where the climate is arid (annual precipitation ranges from 100–160 mm) and the temperature varies from below -30°C in the winter to +40°C in the summer. Because of low seed germination rates in the harsh environments and increasing anthropogenic pressures in their natural range, both *A. mongolicus* and *A. nanus* have been categorized as ‘endangered’ species. As a relict survivor of the evergreen broadleaf forest in this region from the Tertiary period, *Ammopiptanthus* has acquired the strong ability to adapt to the dry and extremely cold environments. The specificities above have recently attracted scientific attention to their anatomy [10], cold and drought stress resistance [11-13], and genetic diversity and geographic differentiation [14,15]. However, little genome information has been reported in regard to its resistance mechanisms to environmental stresses, despite this genotype with strong tolerances making *A. mongolicus* an excellent species to study cold and drought tolerance.

Understanding a plant’s response to a stress will require a global analysis of stress-responsive genes. A significant insight into the expressed portion of a plant with a large size and unknown genome is through large-scale generation and analysis of expressed sequence tags (ESTs). cDNA libraries prepared from different tissues exposed to various stress conditions are valuable tools to obtain the expressed and stress-regulated portion of the genome. This not only leads to the identification of genes that have known or putative functions, but also to the discovery of novel, previously unknown putative genes. Recent studies have demonstrated that the EST approach has been successfully used to discover novel genes and to investigate expression patterns of woody plants in response to abiotic stresses, such as the expression profile of *Tamarix androssowii* in response to NaHCO_3_ stress [16], ESTs of apple (Malus x domestica ‘Royal Gala’) in response to low temperature and water deficit [17], ESTs of poplar leaves subjected to multiple abiotic stresses [18], and ESTs from cold-acclimated and non-acclimated leaves of *Rhododendron catawbiense* Michx [19]. In the present study, more than 5,000 ESTs were generated from *A. mongolicus* seedlings after cold and drought stress treatments, allowing construction of an AmCDUnigene (*A. mongolicus* cold and drought stress unigenes) set of 1,594 putative genes. These data offer a valuable genomic resource to study cold-acclimation and drought adaptation in *A. mongolicus*, and to identify key genes in regulation of cold and drought tolerance in this plant.

## Results

### The quality of the cDNA Library

Since we aimed to identify both early and late cold-responsive and/or drought-responsive genes through EST analysis, a cDNA library, AmCDL (*A. mongolicus* cold- and drought-responsive library), was constructed from pooled samples of *A. mongolicus* seedlings at different stages of cold treatment and water deficit. Blue/white plaque selection following incubation of an aliquot of the library showed a 95.5% recombinant rate with titers of 1.01×10^6^. Insert length was evaluated with 96-randomly selected representative clones by PCR amplification and sequencing before large-scale sequencing. According to the sequencing data, the mean size of the insert cDNAs was 0.8 kb. The full length ratio of the 96 sequenced clones was 57.4% by BLAST searching the 5’end sequences in GenBank.

### Generation and analysis of ESTs

**Figure 1:**
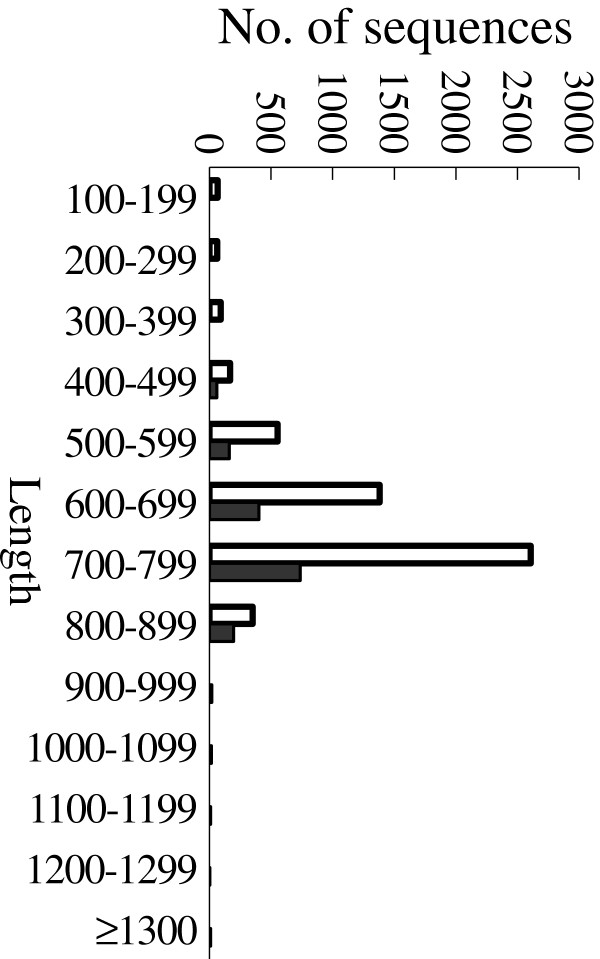
**Size distribution of ESTs and unigenes.** Open bars, all 5282 sequenced ESTs; Filled bars, the 1594 unigene sequences.

The main objective of this study was to produce collections of EST sequences and cDNA clones in order to discover abotic stress-responsive or abiotic stress-tolerance genes in *A. mongolicus*. Bacterial clones were randomly picked from the library and 6,240 (65×96) single pass sequence reactions (IDs SDQ001_A01 to SDQ065_H12) were performed on cDNAs present in plasmid from their 5′ ends of these clones (Table 1). Thus, this cDNA library generated 5,282 high quality ESTs of 100 bp or longer, with an average sequence length of 681.6 bp. The sequence lengths of more than one half (55%) of the ESTs are above 700 bp with the longest sequence being 865 bp (Figure 1). Based on homology searching, 3,741 (70.8%) sequences matched their homologs in the NCBI sequence (non-redundant nucleotide or protein) databases, and 57.5% (3,064) of these inserts represented full-coding lengths (Table 1) based on comparative BLAST analysis.

The 5,282 ESTs were clustered into 461 contigs and 1,145 singletons, giving 1,606 candidate genes (Table 1). After removing rRNA, mitochondrial, chloroplast sequences, and some redundant sequences, the candidate gene set consisted of 1,594 unigenes with an average length of 720 bp and the longest unigene of 1,713 bp (Figure 1). These final genes were denoted as the AmCDUnigene (*A. mongolicus* cold and drought stress unigenes) set.

**Figure 2:**
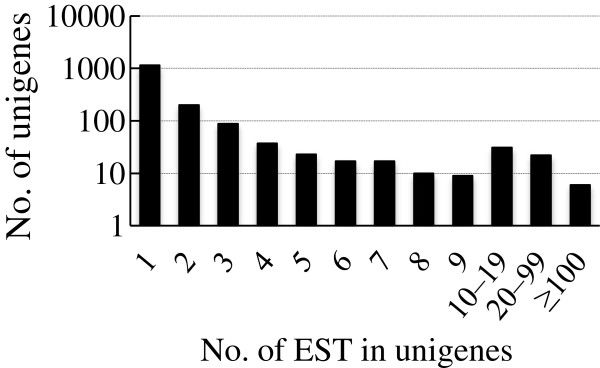
Numbers of ESTs in unigenes.

The EST number per unigene is shown in Figure 2. Of 1,594 unigenes, 1,145 are singletons. Two hundred unigenes contain two ESTs per locus. Twenty-eight unigenes had more than 20 ESTs per unigene, and this group of genes represents 42% of the ESTs. The six largest unigenes (number of ESTs per cluster is larger than 100) represent more than one quarter (27.9%) of the ESTs. As predicted, the number of unigenes containing higher numbers of ESTs decreased as the number of ESTs per unigene increased (Figure 2).

**Table 1:** **General characteristics of *A. mongolicus *ESTs, showing number of ESTs in each category**

	
Total number of readable sequences obtained	5637
Vector sequence, low quality and other contaminants	311
Sequences below 100bp	44
High quality ESTs	5282
Full-length ratio of ESTs (Percentage)	3064 (57.5%)
Contigs	461
Singletons	1145
Candidate genes	1606
ESTs matching to Nr database (Percentage)	3741 (70.8%)
Unigenes matching to Nr database (Percentage)	1123 (70.4%)
Unigenes	1594

### Annotation and functional classification

The annotation of the AmCDUnigene set was based on sequence homology. Each gene in the unigene set inherited the annotation from the best match found after a BLASTX search against the NCBI protein database. Search results revealed that 70.4% of the unigenes had hits with known putative functions, and the remaining 29.6% showed some homology with no characterized function such as unknown proteins, hypothetical proteins, etc. As the ESTs in this study were obtained from a cDNA library constructed from cold- and drought-stressed *A. mongolicus* seedlings, it is possible that some of these 29.6% unigenes were involved in the cold- and/or drought-stress response and/or are unique genes in *A. mongolicus*, though they may be expressed at varied levels under non-stress conditions. However, validation of their function will be the focus of our future studies.

**Figure 3:**
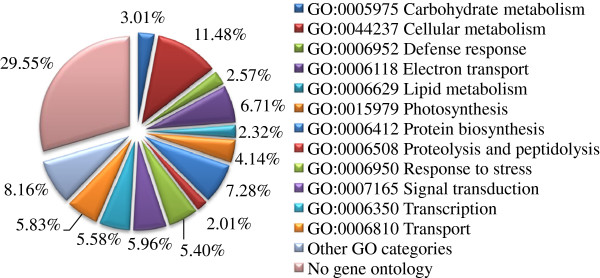
Functional classification of the unigenes in the AmCDUnigene set.

The non-redundant sequences were further analyzed by the assignment of gene ontology (GO) terms. Over 60% of the unigenes were grouped into 12 GO categories. Almost 30% of the unigenes (471 unigenes with *E-*values > 1e-10 in the BLASTX search) could not be assigned and therefore classified as “No GO ontology” (Figure 3). The most

 prevalent GOSlim categories were that of cellular metabolism (11.48%), followed by protein biosynthesis (7.28%) and electron transport (6.71%), suggesting a high degree of basic metabolic activity in the stressed tissues. Less prevalent GOSlim categories were those of response to stress (5.40%), signal transduction (5.96%), transcription (5.58%) and transport (5.83%). These GO categories have been implicated in the general stress response pathways consisting of stress recognition, downstream signaling events, defense and adaptation responses.

### The best matched species

**Figure 4:**
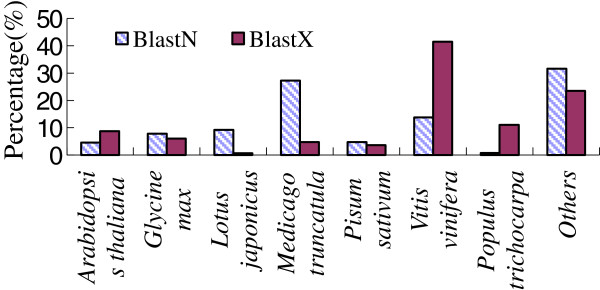
Best matching species of all the unigenes in the AmCDUnigene set in BLAST searches.

While 30% of the unigenes were unknown or hypothetical proteins, almost all of them (95%) matched sequences from other species. Of all unigenes, best hits in BLASTX searches were mainly to *Vitis vinifera* (662 hits, 41.5%), *Populus trichocarpa* (176 hits, 11.0%), *Arabidopsis thaliana* (139 hits, 8.7%), and *Glycine max* (97 hits, 6.1%); best hits in BLASTN searches were mainly to *Medicago truncatula* (434 hits, 27.2%), *Vitis vinifera* (220 hits, 13.8%), *Lotus japonicus* (148 hits, 9.3%) and *Glycine max* (125 hits, 7.8%) (Figure 4). Out of the 1,594 unigenes, only four had a best hit with *A. mongolicus* (0.25%), indicating that little information is available in the genome of this species. Interestingly, more than one half (55%) had best hits with woody plants such as *Vitis vinifera* and *Populus trichocarpa* in BLASTX searches, whereas only 15% had best hits in

 BLASTN searches. Similarly, more than one half (57%) had best hits from the sequences of legume (including *Glycine max, Medicago truncatula, Lotus japonicus, Pisum sativum* and *Arachis hypogaea* etc.) in BLASTN searches and less than one quarter had hits in BLASTX searches. This is reasonable for the legume woody plant *A. mongolicus* considering the bias of codon usage.

### The most abundant ESTs

Apart from providing an efficient method for gene discovery, EST data sets can be used to provide low precision estimates of mRNA levels through estimations of EST redundancy. In this study, the redundancy of specific genes was worked out according to the results of the 5′ annotation (Table 2) with the best homolog match from a BLASTX search against the NCBI protein database. Of the 461 contigs from the AmCDL library, 13 contigs in the 20 most highly represented transcripts encode photosynthetic-related proteins, such as ribulose 1, 5-bisphosphate carboxylase/oxygenase small chain (650 clones), chlorophyll a/b binding proteins (265 clones for type I and 156 clones for type II), plastocyanin (142 clones), and photosystem reaction center subunits (Table 2). The other abundant transcripts included metallothionein type 2 (134 clones), lipid transfer protein-like protein (70 clones), auxin-repressed protein, carbonic anhydrase, and ferredoxin-1.

ESTs abundance was further analyzed based on the classification of corresponding unigenes. Less than 4% photosynthetic genes were derived from 28% ESTs. Electron transport genes (6.71%, Figure 3) were also abundant according to the ESTs classification percentage (6.17%). This category included multiple electron transport genes that were involved in radical scavenging or detoxification, such as the abundant transcripts of metallothioneins, ferredoxins and thioredoxins.

### Identification of stress-responsive genes

A large number of genes in the AmCDUnigene set could be identified as stress-responsive genes or defense genes

**Table 2:** **Putative function of the 20 most abundant sequences in cold- and drought-treated *A. mongolicus *seedlings**

**Contig**	**Number of ESTs**	**Annotation**	**Accession**	***E-*value**
AM0003	650	Ribulose-1,5-bisphosphate carboxylase	AAA81328	7e-82
		small subunit rbcS1		
AM0084	265	Chlorophyll a/b-binding protein type I	AAA50172	1e-117
AM0022	156	Chlorophyll a/b binding protein type II	AAL29886	1e-139
AM0128	142	Plastocyanin	P17340	3e-53
AM0044	134	Metallothionein type 2	ABQ44281	9e-24
AM0016	125	Chloroplast photosystem II PsbR	ABW35320	1e-58
AM0244	70	Lipid transfer protein-like protein	AAL32039	3e-40
AM0132	64	Photosystem I reaction center subunit XI	ABD28376	1e-89
AM0001	45	Glycine cleavage system T protein	P49364	1e-136
AM0014	45	Photosystem I reaction center subunit X	AAP03873	2e-56
AM0086	42	Carbonic anhydrase	AAD27876	1e-108
AM0021	37	Auxin-repressed protein	ABQ44282	6e-37
AM0133	33	Ferredoxin-1 precursor	AAB61593	1e-38
AM0307	32	No hit		
AM0011	29	Chloroplast oxygen-evolving enhancer protein	AAV74404	6e-82
AM0042	28	Rribulose-1,5-bisphosphate carboxylase	ABB20913	1e-122
		/oxygenase activase alpha 2		
AM0038	27	Pore protein	CAA97910	2e-61
AM0070	24	Ribulose-5-phosphate-3-epimerase	AAM19354	1e-123
AM0445	24	Photosystem I psaH protein	ABI84258	5e-47
AM0764	24	Photosystem I reaction center subunit III	BAF80474	2e-43

 according to the earlier GO categories, but the functions of other genes involved in stress responses remain to be determined. Comparative *in silico* analyses of all *A. mongolicus* unigenes with those in other plants led to the identification of a putative function for 528 stress-responsive genes. These genes account for 33% of the total unigenes and 30% of the ESTs, which were classified into 12 functional groups (Figure 5) based on similar functional characteristics, cellular roles or their involvement in cellular processes or pathways. The list of the 528 stress-responsive genes identified from our ESTs, along with the annotation and GO category is presented as supplementary file, see Additional file 1.

Interestingly, the most abundant category is “electron transport” (76 unigenes, 14.39%), which includes 16 thioredoxins, 9 ferredoxins, 7 glutaredoxins, 5 metallothioneins, 4 superoxide dismutases and multiple other genes that participate in reactive oxygen species (ROS) scavenging. The most abundant products of this category indicated the importance of cell redox homeostasis in cold- and drought-stressed *A. mongolicus* seedlings. The categories of “response to stress” (61, 11.55%) and “transport” (65, 12.31%) are also abundant groups in these stress-responsive genes. The category of “response to stress” contains various protein genes, encoding multiple cold regulated proteins (CORs), dehydrins, late embryogenesis abundant proteins (LEAs), 14-3-3 proteins, glyoxalases betaine-aldehyde dehydrogenase (BADH), which may be involved in multiple metabolic pathways and most of which act as protectants to prevent macromolecular coagulation and injury of membrane structure. The abundance of the transport group suggested that this system was active under cold and/or drought conditions. The transcripts of several abundant proteins were highly present in the transport category, such as 3 aquaporins, 4 ATP-binding cassette (ABC) transporters and 8 lipid transfer proteins, etc., which may be able to carry various substrates, including water, ions, carbohydrates and lipids in the plant system.

**Figure 5:**
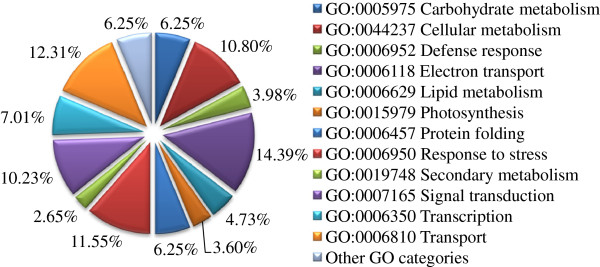
Functional classification of 528 putative stress-responsive genes in the AmCDUnigene set.

Additionally, to ascertain whether these stress-responsive genes were involved in the response to cold or drought stress in *A. mongolicus*, we compared these stress-responsive genes with previously reported abiotic stress genes from other organisms documented in the Comparative Stress Genes Catalog of GCP (Generation Challenge Programme, http://www.generationcp.org). This web site has compiled a comparative crop stress gene catalog with available information of four plant abiotic stresses (cold, salt, ABA and drought). Surprisingly, only 120 abiotic stress-responsive genes were found in 78 families in the Catalog (Additional file 2: Table S1, Additional file 3). These genes were involved in the ROS scavenging, signaling cascades, transport, membrane composition, photosynthesis, transcription regulation and protective functions. Interestingly, 90% of families (50/55) were identified as drought-responsive genes and only five of them were cold-responsive (Additional file 3), except for 23 families whose stress types were not shown in the Catalog. More genes responded to drought than cold, indicating that drought-responsive genes were dominant in cold- and/or drought-stressed *A. mongolicus*. Furthermore, the EST number of the 120 (7.5%) contigs account for 16.6% of the total ESTs, suggesting the abundance of these genes in *A.mongolicus*. Multiple abundant genes with more than ten ESTs were present in these genes, including 6 of the top 20 most abundant unique sequences (Table 2, Additional file 2: Table S1). They mainly participate in the photosynthesis and oxidoreduction pathways.

### Differential expression of cold- or drought-inducible genes in *A. mongolicus*

To further characterize abiotic stress-responsive genes listed in Additional file 3 as cold- and/or drought-inducible genes identified in *A. mongolicus*, the expression pattern of the 120 genes under cold and drought conditions were analyzed by qRT-PCR. Relative transcription values of 97 genes were obtained (Additonal file 3). Their fold changes in expression of the treatments relative to that of the control at each time point were color coded and shown in Additional file 2: Table S1. Some uniscripts with abundant ESTs in Table 2 were noted for being highly expressed as shown in Additonal file 3, which confirmed the quantitative EST results. It was obvious that many of the uniscripts had strongly enhanced expression under cold and/or drought conditions; however, the differences in the response pattern between the two treatments were striking, suggesting that these genes may play different roles in *A. mongolicus* under the two types of stresses. Despite several attempts using different primer pairs, we could not obtain reproductive expression patterns of 23 transcripts, thus they were shown as blank in Additional file 3.

The qRT-PCR results confirmed that 85% of the abiotic stress-responsive genes (82 of the 97 detected uniscripts) were cold- or drought-inducible in *A. mongolicus.* Most of these inducible genes (76 of 82) were up-regulated under the cold condition. Less than half (47, 48%) of these detected genes were up-regulated under the drought condition. Moreover, compared with 35 unigenes expressed specifically under cold treatment, only 7 genes were expressed uniquely under drought treatment. More genes appeared cold-inducible than drought-inducible in *A. mongolicus*, which is not consistent with the display of stress type in the Catalog. These inconsistencies may be attributed to the biological differences among plant species used, stress treatment conditions, their response to abiotic stresses, and/or detection methodologies. Since more cold-inducible genes were found in this study, it may be suggest that cold stress or low temperature is a more severe limiting factor for *A. mongolicus.*

Among these stress-induced genes, we found that five genes showing significant induction (≥8 fold) in at least one time point during cold or drought stress were well known stress-inducible genes, including NAC domain protein gene (AM0328), actin depolymerizing factor gene (AM0262), beta-amylase gene (AM0598), low temperature and salt responsive protein gene (AM0047), and ubiquitin-conjugating enzyme E2 gene (AM0976). Several transcripts displayed exclusive or predominant stress specificity, for example GTP binding protein RAB1D gene (AM0790) and actin depolymerizing factor gene (AM0262) for drought stress, but NAC domain protein gene (AM0328), desiccation protectant protein Lea14 gene (AM1436), low temperature and salt responsive protein gene (AM0047) and chloroplast Cu/Zn SOD gene (AM0701) for cold stress. Little is known about the function of these genes under cold and/or drought stresses in *A. mongolicus,* though some genes were found responsive to abiotic stress in other species. The action mode of these genes in *A. mongolicus* will be further studied in our lab.

### Identification and characterization of SSRs

A set of 1594 unigenes sequences, comprising a total of 1.13 Mb of sequence, was searched for SSRs with a repeat motif length ranging from two to six nucleotides using SSIT. In total, 155 di- to hexa- nucleotide SSRs were identified from 138 (8.7%) unigenes (Additional file 4), with an average of one SSR per 7.2 kb. Of those, 16 (11.6%) genes contained more than one SSR. Analysis of these SSR motifs revealed that the proportion of SSR unit sizes was not evenly distributed. Most of these satellites are di- or tri- nucleotide motifs, being 36 (23.2%) and 76 (49%). The occurrences of tetra- to hexa-nucleotides were 19 (12.3%), 9 (5.8%) and 15 (9.7%), respectively. The AG/CT was the most frequent repeat motif and accounted for 10.3% (16/155), followed by AAG/CTT (7.7%, 12/155), AGA/TCT (7.7%, 12/155), GAA/TTC (6.4%, 10/155) and AG/CT (5.8%, 9/155). Most of the tetra- to hexa-nucleotide motifs were found only once. The mean SSR length of each unit varied between 14 and 30 bp. The overall average of SSR length was 19 bp with a maximum of 36 bp hexa-nucleotide repeat (AACCCA).

Table 3 shows the type and position of SSRs in the gene sequences. The majority (68%, 105/155) of the identified SSRs are present in the untranslated regions (UTRs), including all dinucleotide, tetrancleotide and pentanucleotide repeats. Trinucleotide (57%, 43/76) and hexanucleotide (47%, 7/15) repeats, causing no frame-shift, are mainly found in the coding regions. In contrast, repeat motifs containing di-, tetra- and pentanucleotides, resulting in frame shifts when undergoing slip-strand mutations, were primarily found in untranslated regions, and rare in ORFs. And SSRs in the 5′ UTR (71) are more frequent than in the 3′ UTR (23).

Additionally, among the 138 unigenes containing SSRs, 53 (38%) were stress-responsive genes and 37 (27%) were unknown. These stress-responsive genes include ten proteins that participate in radical scavenging, such as thioredoxin, cytochrome C reductase, glutaredoxin and peroxiredoxin, etc., five signal transduction proteins (SOS2, calmodulin, serine/threonine phosphatase, etc*.*), nine stress-responsive proteins (dehydrins, stress enhanced protein, cold acclimation responsive protein and defensin), nine transcription factors, four heat stress proteins and secondary metabolism proteins, etc.*.* The most frequent motifs contained in these stress-responsive genes were AAG/CTT, AG/CT and AGA/TCT. Interestingly, more than one half of SSRs were present in the 5′UTR of the 53 stress-responsive genes, one third within ORFs and only five in the 3′UTR.

## Discussion

ESTs have proven to be a valuable tool for gene discovery and a number of EST collections from many different plants are now publicly available according to the Expressed Sequence Tags Database in NCBI (http://www.ncbi.nlm.nih.gov/dbEST). In *A. mongolicus*, an ideal plant for investigating the cold and drought resistance mechanisms of the woody plants endemic to the desert of northwestern China, only hundreds of ESTs have been sequenced and a small number of sequences from cold- or drought-treated seedlings are available in the NCBI databases despite a recent report on a transcriptomic study with PEG-treated roots of this species [20]. Therefore there is a great need for developing more ESTs on abiotic stress genes of this shrub. In this study, we obtained 5,282 sequences from cold- and drought-treated seedlings of *A. mongolicus*, giving 1,594 unigenes. Since we are interested in all the genes involved in abiotic stresses, leaves treated from short term (1 h) to long term (8 d) were pooled for cDNA construction and EST generation. Molecular functional classification of the 1,594 unigenes shows a large number of genes that are predicted to be involved in electron transport, response to stress, signal transduction and regulation of transcription. Using comparative analysis of stress-responsive genes from other plants, 528 (33% of total unigenes) (Additional file 1) putative stress genes were identified in the AmCDUnigene set. By comparing the 528 stress-responsive genes with reported abiotic stress genes from other organisms in the Comparative Stress Genes Catalog of GCP, 120 abiotic stress-responsive genes in *A. mongolicus* seedlings were identified, and their transcriptional patterns under cold and drought stresses were characterized by qRT-PCR. Eighty-five percent of detected genes (82 of 97 detected genes) were found cold- or drought-inducible, confirming the AmCDUnigene data in some cases. These genes could be involved in multiple pathways, such as the reactive oxygen species network, photosynthesis, stress response, transcriptional regulation, signal transduction, etc., which probably all participate in stress tolerance improvement of *A. mongolicus*. The putative functions of these pathways and involved genes during cold and drought responses are discussed later.

### Photosynthesis

As leaves were used as the RNA source, it is reasonable to find that the most common ESTs in our collection represent various genes involved in photosynthesis, like ribulose 1, 5-bisphosphate carboxylase/oxygenase small

**Table 3:** Distribution of SSRs with respect to putative open reading frames (ORFs)

**Repeat motif**	**Within ORF**	**5′UTR**	**3′UTR**	**3′mix**	**ORF undefined**	**All**
Dinucleotide	0	29	5	0	2	36
Trinucleotide	41	20	7	2	6	76
Tetranucelotide	0	8	9	0	2	19
Pentanucleotide	0	7	1	0	1	9
Hexanucleotide	7	7	1	0	0	15
Total	48	71	23	2	11	155

UTR, untranslated regions; 3′mix, SSRs extend over stop codon of coding region and UTR.

 chain, chlorophyll a/b binding protein, plastocyanin, photosystem reaction center proteins, and carbonic anhydrase. Some of them were identified as cold and/or drought stress genes (Additional file 2: Table S1). The qRT-PCR results in Additional file 2: Table S1 and Additional file 3 confirmed their abundance and responses to the stresses.

The over-representation of ESTs encoding chlorophyll a/b binding proteins (156 ESTs for type II and 265 ESTs for type I CARCAB1) and their high expression levels and up-regulation detected by qRT-PCR (AM0022 and AM0084 in Additional file 2: Table S1 and Additional file 3) under the cold conditions suggest their participation of cold tolerance of *A. mongolicus.* The enzymes ribulose-1,5-bisphosphate carboxylase/oxygenase activases (AM0042) were also found highly transcribed and up-regulated in cold-treated seedlings (Additional file 2: Table S1 and Additional file 3). Carbonic anhydrase, which is known to maintain optimal CO_2_ concentrations [21], may be thus helping to efficiently use the CO_2_ and available light energy under cold and drought conditions. Expression changes of these genes may reflect their involvement in the stress acclimation of *A. mongolicus*.

### ROS network

Low temperature and water deficit disrupt photosynthesis and increase photorespiration and thus cause an increased production of reactive oxygen species (ROS). ROS accumulation in stress cells must be kept tightly under control by an efficient and versatile scavenging system to avoid damage and cell death. In the cold- and drought-stressed seedlings of *A. mongolicus*, a battery of scavenging enzymes genes were present, including multiple superoxide dismutases (SODs), thioredoxins (Trxs), ferredoxins (Frxs), glutaredoxins (Grxs), peroxiredoxins (PrxRs), metallothioneins (Mts), ascorbate peroxidases (APXs), catalase (CAT), glutathione peroxidases (GPXs), etc., all of which may be involved in the ROS network of acclimation and stress response.

In chloroplasts, not only SODs, but PrxRs and Trxs can provide antioxidative protection for detoxification of photochemically produced H_2_O_2_[22] and play an important role during drought and oxidative stress [23,24]. Trx and Grx are ubiquitous disulfide reductases that regulate the redox status of target proteins, such as peroxiredoxins [25]; GPX display peroxidase activity preferentially in the presence of Trx [26,27]. Trxs also interact through a disulfide bridge with SODs and germin-like proteins [28,29], as well as Frxs [30]. Frx and Grx function in maintaining the reduced state of antioxidants ascorbic acid and glutathione [31]. Additionally, Mts are ubiquitous Cys-rich proteins known to be involved in ROS scavenging [32,33]. The regeneration of Mts after oxidative modification may involve endogenous dithiols, such as Trx [34]. Up-regulation (Additional file 2: Table S1) and abundance of these scavengers in the AmCDUnigene set and the reported above interaction between them indicate the complex and redundant ROS scavenging pathways, which may play a central role in the consequent regulation of acclimation and stress tolerance in cold- and drought-treated *A. mongolicus* seedlings.

### Molecular signals and their roles in transcription

A general stress signal transduction pathway starts with signal perception, followed by the generation of second messengers, which modulate intracellular Ca^2+^ levels, often initiating a protein phosphorylation cascade that finally targets proteins directly involved in transcription factors controlling specific sets of stress-regulated genes [35]. Our data showed that multiple genes were present in the AmCDUnigene set encoding proteins involved in stress signaling cascades, such as different types of protein kinases and phosphatases, Ca^2+^ messenger system proteins as well as GTP binding proteins and ethylene signaling protein. The expression data in Additional file 2: Table S1 showed that GTP binding protein gene (AM1171), calmodulin gene (AM0287) and calcium-dependent protein kinase CDPK gene (AM0463) were greatly increased under both cold and drought conditions, reflecting the multiplicity of cold and drought stress signals in *A. mongolicus.*

The potentially increased activities of various signaling pathways are associated with differential expression of many families of transcriptional factors. The functional class of the transcription associated genes comprised families of transcriptional factors including multiple types of zinc finger proteins (such as C3HC4, C2H2, GATA and AN1), MYB, NAC domain proteins, bZIP transcription factors, AP2 domain-containing transcription factors, and bHLH transcription factors. Several members of these families were previously identified as being responsive to various stresses [36], such as the AP2 family to cold and drought, bZIP to drought and ABA, MYB to dehydration, zinc finger to cold and drought, bHLH and NAC to drought, salinity and ABA. Similar results were obtained in cold- and drought-stressed *A. mongolicus* in which expression of NAC domain protein gene (AM0328) and ethylene response factor gene (AM1353) were significantly up-regulated (Additional file 2: Table S1). An important and noteworthy observation was that zinc finger, bZIP and MYB transcription regulators showed predominance in this plant*.* Though the most studied members are those of the AP2/ERF family, which had previously shown to be the regulators of the majority of cold- and drought-responsive genes, only three contigs encoding ERF, DREB and TINY-like protein were found in this AmCDUnigene set belonging to this family.

### Cellular transport and homeostasis

Stress-induced reorganization and spatial distribution of many key metabolites in plants require efficient transport machinery. Water transport is of great importance to water homeostasis in plants under cold and drought stresses. Up-regulated transcription of aquaporin and membrane intrinsic proteins in *A. mongolicus*, like in a number of other plant species [37], may act as a major player to respond to cold or drought stress. The role of nutrients such as nitrogen and phosphorus on cold hardiness has received attention because low temperatures not only limit nitrification [38], but also inhibit photosynthesis and consequently reduce inorganic phosphate availability [39]. The increased transcription of amino acid transporter (AM1518) and phosphate transporter (AM1013) during cold and drought treatments may indicate a readjustment of the cellular Pi and N status for reestablishing cell nutrient homeostasis in *A. mongolicus.*

It was reported that dehydration and cold stress induced sugar transporters in *Arabidopsis*[40] and sugarcane [41], which may be a response to an increased demand for carbohydrates to protect cells under stress conditions. The non-specific lipid transfer proteins were also reported to respond to drought and in some cases to cold [42,43] by facilitating the transfer of phospholipids, glycolipids, fatty acids and steroids between membranes. Overexpression of one member of the ABC-transporters improved drought and salt stress resistance in *Arabidopsis*[44]. Various transport-associated genes were found in this study, encoding ABC-transporters, non-specific lipid-transfer proteins, sugar transporters, metal transporter, phosphate transporter and nitrate transporter, which may constitute the transport machinery of *A. mongolicus* under cold and drought conditions.

### Protein metabolism

Cellular processes like translation, post-translational events and protein degradation are crucial for cell survival under varied environmental conditions. In the present study, these classes constituted about 15% of the unigenes and 8.9% of ESTs and comprised predominantly of the ribosomal protein genes and heat stress protein genes apart from proteinase genes and ubiquitin genes. Genes encoding multiple ribosomal protein subunits, multiple protein translation initiation factors, and translation elongation factors were all found in this AmCDUnigene set. These proteins play a pivotal role in translation and their presence in the set indicates they play a role in stress response of *A. mongolicus*.

Ubiquitin and proteasome-mediated protein degradation plays a crucial role in enabling plants to alter their proteome to maximize their chances of survival under various circumstances [45]. Expression data (Additional file 2: Table S1) showed six ubiquitin-conjugating enzymes were all significantly up-regulated in *A. mongolicus* during cold and drought stresses, suggesting their involvement in stress tolerance of this plant.

It is well established that molecular chaperones, often called heat shock proteins, are critical for cell survival in times of stress to ensure proper folding of protein substrate, protein disaggregation and protein degradation. Five of six major families of heat shock protein genes in plant are presented in this AmCDUnigene set, including Hsp70 family (DnaK/Ssa), J-protein/Hsp40 family (DnaJ/Ydj1), Hsp90 family, Hsp100 (caseino-lytic proteinases, Clp) family and small Hsp family (sHsp). A current model for sHsp function proposes that they protect thermo-sensitive substrates from irreversible heat stress-induced denaturation and aggregation [46]. J-proteins not only play a crucial role in maintaining the protein homeostasis by reestablishing functionally native conformations but are involved in stress signaling pathways during various abiotic stresses [47]. The major functions of Hsp70 were also multiple, such as preventing aggregation, assisting refolding, protein import, signal transduction and transcription activation [48]. The transcription of these Hsps/chaperaones and improved transcription of Hsp70s (AM0676 and AM0734) and Hsp90 (AM0898) (Additional file 2: Table S1) suggest that similar protein protection mechanisms exist in *A. mongolicus*.

### Carbohydrate metabolism

Some genes that are involved in carbohydrate metabolism and up-regulated under stress conditions in other plants were also found up-regulated in cold- and/or drought-stressed *A. mongolicus* (Additional file 2: Table S1). Most of these genes regulate starch and sugar metabolism and may participate in the protection of macromolecules in cells from damage. For instance, beta-amylase, which has a primary role to produce maltose during hydrolytic starch degradation and was up-regulated under cold conditions [49] in *Arabidopsis*, was significantly up-regulated under both cold and drought conditions in *A. mongolicus*. The expression of trehalose-phosphatase/synthase (AM1068) was increased according to qRT-PCR assay as presented in Additional file 2: Table S1, indicating its involvement in drought stress tolerance of *A. mongolicus*. Similar results were reported in transgenic *Arabidopsis* where overexpressing *AtTPS1* (trehalose-6-P synthase) displayed a dehydration tolerance phenotype [50], and *AtTPS5* was also found in the involvement of stress tolerance [51].

### Plant responses to stress

The AmCDUnigene set represents a rich source of stress-responsive *A. mongolicus* genes and presented multiple dehydration-responsive protein genes, late embryogenesis abundant (LEA) protein genes, and cold-regulated protein genes. Usually, dehydration-responsive protein genes respond to drought and cold-regulated protein genes express under cold, though some of them respond to more than one stress. For instance, desiccation protectant protein *Lea14* (AM1436) is up-regulated not only under drought but also cold stress, which is similar with other LEA protein genes accumulating in vegetative tissues exposed to dehydration, osmotic, and low temperature stress [52,53]. LEA proteins have been proposed to stabilize membranes and prevent crystallization of cellular components due to their extreme stability and hydrophilicity [54]. The uningene (AM0047) encoded a protein that is similar to hydrophobic protein RCI2A (RARE-COLD-INDUCIBLE 2A), also known as low temperature and salt responsive protein, and its expression was highly promoted by cold and drought treatments. In general, RCI2 genes could be induced by ABA and abiotic stress, and were potentially involved in the regulation of plasma membrane potential [55]. The up-regulated *LEA* and *RCI2A* in *A. mongolicus* may illuminate their participation in cell protection and rescue processes.

### Defense response

Defense-related genes encoding proteinase inhibitors, plant defensins, disease resistance proteins and chitinases were found in the AmCDUnigene set. Many disease resistance protein genes have shown to be expressed in response to abiotic stresses [56], but their exact role remains unknown. The chitinase genes are also up-regulated by abiotic stress such as drought [57], and by ethylene and jasmonic acid [58], though their biological role under drought remains unclear. Jasmonic acid was reported to significantly affect expression of drought-related genes, indicating that there is extensive cross-talk between responses to drought and other environmental factors including biotic stresses [59]. The presence of these defense response genes in *A. mongolicus* implicates their involvement in abiotic response besides their key role in pathogen stress.

### Unkown genes

About 30% of the 1594 unigenes genes were identified with unknown functions. These were divided into two groups, one in which homologous or similar genes from other organism exist and one where no significant similarities could be found to any other sequence, i.e. genes that could be *A. mongolicus*-specific. Assuming that many of these 471 *A. mongolicus*-specific unknown genes are cold- and/or drought-related, some new abiotic stress genes may be present in this collection. Such genes are potentially interesting and could encode unknown proteins or regulatory factors that need to be characterized to discover new pathways and mechanisms adapted by plants to cope with abiotic stresses. A study is necessary for the 471 genes to elucidate which *A. mongolicus*-specific unknown genes are responsive to cold or drought stress.

### SSRs

SSRs derived from ESTs essentially represent expressed genetic sequences and hence are potential candidates for the construction of markers for gene tagging and comparative genomic studies [60]. In this study we found trinucleotide repeats to be the most common SSR type in ESTs of *A. mongolicus.* This is in agreement with a majority of studies that report trinucleotide repeats were the most abundant class of SSRs in *A. mongolicus* sequences [20] and in other plant ESTs [61]. Interestingly, more than one half of known unigenes that contain SSRs were stress-responsive genes with SSRs present in the 5′UTR or within ORFs, which indicated that these SSRs may be involved in regulating expression of these genes or enhancing protein functions. The exact function for these SSR-containing stress genes and how they occurred need to be further characterized. Thus, characterization of these stress-responsive genes and gene-based functional markers may provide a shortcut to illuminating the mechanism of stress tolerance requirement in *A. mongolicus.*

## Conclusion

The AmCDUnigene set containing 1,594 genes was generated from a cDNA library constructed from *A. mongolicus* seedlings subjected to cold and drought stresses. Of these, 30% of unigenes encoded unknown function proteins and 70% of unigenes were annotated and classified into 12 broad functional categories. Further analysis revealed that 528 (33%) unigenes had similarity to genes known to be involved in stress responses in other plant species such as ROS scavenging, cellular transport, signal transduction and transcription processes. By comparing the 528 stress-responsive genes with reported abiotic stress genes in the Comparative Stress Genes Catalog of GCP, 120 abiotic stress-responsive genes in *A. mongolicus* seedlings were identified. Transcriptional analysis showed that 82 genes were cold- or drought-inducible, confirming that ROS network, signal transduction and osmolyte accumulation undergo transcriptional reorganization when exposed to cold or drought stress. Additionally, 155 SSRs were found in the 1,594 unigene sequences.

This study represents an exploration of molecular responses of *A. mongolicus* seedlings to cold and drought stresses using trancriptomic analysis. Together, the abiotic stress-responsive ESTs and the subsequent gene expression analysis provide a large number of excellent candidate genes for future investigation into the adaptation of *A. monglicus* in adverse conditions. However, a better understanding of the biological function of those stress-responsive genes and the genetic mechanisms underlying the stress tolerance of *A. mongolicus* would be anticipated in the future studies using an integrated approach involving physiological evaluation and experimental validation. In addition, the genomic resources and advanced knowledge developed from this study will contribute to efficient breeding of new trees and ornamental woody plants with improvement in abiotic stress tolerance.

## Methods

### Plant materials and growth conditions

Two-week-old *A. mongolicus* seedlings were used in this work. Seeds were obtained from an experimental forest farm in Dengkou of Bayannaoer in Inner-Mongolia of China. The seeds were germinated in pots with water-saturated medium and maintained in growth conditions of 60% relative humidity under an illumination of 200 μmol m^-2^ sec^-1^ and a 14-hour photoperiod with 26°C during the day and 22°C during the night. Cold treatments were performed by incubating the seedlings at 4-6°C for 1 h, 2 h, 4 h, 8 h, 24 h, 48 h, 96 h and 8 d, then the aerial parts were sampled at each time point and pooled. For water deficit treatment, seeds were sown in the same medium to germinate in growth conditions as described above, and then no water was added to the medium. It was the fifth day when 80% of the seedlings were wilting (seedlings showing 50-60% relative water content and medium showing 50% field capacity) due to lack of water. Samples were harvested at the last four days (i.e., the 12th, 13th, 14th and 15th days after sowing seeds) of the 15-day drought treatment period, respectively (50-70% field capacity). Seedlings of *A.mongolicus* prepared similarly but without any treatment served as the control (CK) in this study.

### RNA isolation, cDNA library construction and EST sequencing

The samples of every time point were pooled in equal amounts. Total RNA was extracted from the pooled seedlings as described by Chang *et al.*[62] with some modifications. Frozen tissues were ground to a fine powder in liquid nitrogen, transferred to 68°C CTAB extraction buffer, and extracted twice with phenol:chloroform (1:1) in equal volumes. RNA was precipitated overnight at 4°C with 2M lithium chloride. The precipitate was dissolved in 0.5% SDS, extracted with an equal volume of chloroform, precipitated with two volumes ethanol, and re-suspended in DEPC-treated water. Poly(A)^+^ mRNA was isolated from total RNA using the Oligotex Direct mRNA kit (Qiagen, Valencia, CA, USA) according to manufacturer’s instructions and was quantified spectrophotometrically at OD260.

The cDNA library was created using SMART™ cDNA Library Construction Kit (Clontech, Palo Alto, CA, USA). The SMART™IV Oligonucleotide was used in the first-strand synthesis to generate a high yield of full-length, double-stranded cDNA. Synthesized cDNA was digested with asymmetrical *Sfi*I restriction enzyme sites at the 5′ and 3′ cDNA ends and ligated into *Sfi*I-digested vector pBluescript II SK, which was modified and contains the asymmetrical *Sfi*I sites in the MCS and facilitates directional cloning. White clones grown on screening LB medium (Amp/IPTG/X-Gal) were recovered by random colony selection and inserts were sequenced from the 5′ end using T7 primer and an ABI 3730 capillary sequencer in Shanghai United Gene Institute of China.

### Sequence processing and analysis

Standard sequence processing tools, Phred [63], Phrap and Cross_match [64,65], were used with Codoncode InterPhace (http://www.codoncode.com). The vector sequence, low-complexity, repeats, poly (A) and low quality regions present at the beginning and end of each sequence were trimmed using Phred 20 cutoff value. Vector screening was performed using the Cross_match program with Codoncode InterPhace software. Sequences were edited for the removal of rRNA, mitochondrial DNA and other contaminants. In the next step, PHRAP was used to assemble the individual ESTs into clusters of sequences derived from the same transcript to reduce the redundancy of the dataset and increase the overall quality of the derived consensus sequences. The criterion setting for clustering sequences together was an alignment of a minimum of 100 bases and with at least 90% similarity between the aligned sequences. Sequences that did not fit into a contig were defined as singletons. Finally, ESTs in singletons that had passed the filters and unmasked sequences < 100 bases were discarded. The resulting singletons and contigs represented the AmCD (*A. mongolicus* cold and drought) candidate gene set.

### Function annotation and classification

Various genomic tools were used to identify annotated sequences. Homology searches were performed against non-redundant nucleotide and protein sequence databases using BLASTN 2.2.12 and BLASTX 2.2.12 versions of the BLAST programs through BLAST 2.0 network client software. The accession numbers and *E*-value of the best matches were extracted from the result files. If two or more query sequences resulted in best matches with identical accession numbers, they were sorted according to their *E*-values. Only the sequence with the lowest *E-*value was included in the unigene set. The unigenes were annotated based on the results of BLASTX searches of the non-redundant database. The definition line of the BLAST match was used as a description of the putative function of the unigene. An *E-*value threshold of 1e-10 was used and unigenes that did not meet this requirement were annotated as unknown. Assigning GO (Gene Ontology) category to each of the unigenes was based on this annotation.

### Identification of genes associated with abiotic stress

The genes associated with stress responses were identified from multiple sources, based on the complied list of stress-regulated genes documented in various plant species (http://stress-genomics.org). Data from microarray expression profiles and ESTs of possible abiotic stress genes in *Arabidopsis*[1,2,48], rice [3,4,66], barley [67] and other plants [40,68] were also used. The unigenes were also compared with those abiotic stress response genes in other organisms reported by the GCP (Generation Challenge Programme) at the Comparative Stress Genes Catalog website (http://dayhoff.generationcp.org) for candidate abiotic stress genes in *A. mongolicus*. The accession numbers in UniProt of the best BLASTX matches with *E*-value < 1e-10 were used to search in the Comparative Stress Genes Catalog.

### Quantitative real-time PCR (qRT-PCR)

The candidate abiotic stress genes from ESTs were validated by performing qRT-PCR at each of the 4 time points of cold treatment (1 d, 2 d, 4 d and 8 d), drought treatment (12th, 13th, 14th and 15th days after sowing, which were referred as 1 d, 2 d, 3 d and 4 d of treatment) and control, respectively. Total RNA from each time point and control were treated with RNase-free DNase to remove residue DNA. Two micrograms of total RNA were reverse-transcribed using 1 μL 50 μM oligo (dT)_20_ and 200 units of SuperScript^TM^ III Reverse Transcriptase (Invitrogen, Carlsbad, CA, USA) following the manufacturer’s instructions. Triplicate qRT-PCR reactions were performed in 384-well plates with ABI PRISM7900HT Real-Time PCR System (Applied Biosystems, CA, USA) using the SYBR^**R**^*Premix Ex Taq*^*TM*^ kit (Takara, Japan), forward and reverse (final concentration 0.2 μM) primers and 100 ng cDNA template. The cycling program was: 95°C for 30 s, 40 cycles of 95°C for 5 s and 60°C for 34 s. PCR primers were designed using Primer Premiers 5.0 and had a primer length of 20–24 nucleotides, a GC content of 40-60% and an optimal annealing temperature of 58 to 60°C, with amplicon lengths between 100 and 250 bp. Triplicate measurements were averaged and the mean value was used for further calculations. Melting curve analysis was performed after PCR to evaluate the presence of any nonspecific PCR products and primer dimers. Genes relative transcription levels were normalized to the geometric mean of two EIF (eukaryotic translation factor) genes, *eIF1* (JN885965) and *eIF3* (JN885967) [69]. The fold change for a particular target was determined by comparing the relative transcription value of the treatment with that of the control. A statistical cut-off, *P* <0.05, was used to determine which genes were differentially expressed. The probe sets and the corresponding primer pairs used for the analysis are listed in Additional file 5.

### Identification of SSRs and open reading frame

The tool of SSRIT (Simple Sequence Repeat Identification Tool) online (http://www.gramene.org/db/markers/ssrtool) [70] was used to search for SSRs (simple sequence repeats) with a repeat motif length of two to six nucleotides sequences in the 1,594 candidate genes set. The minimum repeat unit was defined as seven for di-nucleotides, five for tri-nucleotides and four for tetra-, penta-, and hexa-nucleotides, according to previously described by Bräutigam *et al.*[71]. Compound SSRs were defined as ≥ 2 SSRs interrupted by ≤ 50 bases [72].

To predict the position of SSRs with respect to coding regions, the open reading frames (ORFs) were identified. ORF prediction was based on BLASTX hits and ORF finder tools (http://www.ncbi.nlm.nih.gov/gorf/orfig.cgi). The part of the ORF that matched the best BLASTX hit was considered as seed to select the right coding region from the six frame translations provided by ORF finder. Based on the both results, we defined the beginning and the end of the ORF. Usually an open reading frame starts with an ATG (methionine) and ends with a stop codon (TAA, TAG or TGA).

### Supporting data

The sequencing data have been submitted to the dbEST of GenBank under the accession numbers of JZ356671 - JZ356878, JZ388729 –JZ389985 and the library accession number of LIBEST_028198, *Ammopiptanthus mongolicus* cold- and drought-responsive library.

## Competing interests

The authors declare that they have no competing interests.

## Authors’ contributions

MQL performed EST data analysis and contributed by planning, supervising and financing the work and with writing the paper. MQL and JS carried out cDNA library construction and qRT-PCR experiments from plant material preparation through data analysis. CFL contributed with EST data analysis. All authors read and approved the final manuscript.

## Supplementary Material

Additional file 1 A list of 528 stress-responsive genes with annotation and GO category. Click here for file

Additional file 2 **Table S1.** Change in expression of 97 abiotic stress-responsive genes in A. mongolicus seedlings subjected to cold or drought stress. Values were color coded to represent the fold changes in expression of treatments relative to control at each time point. Significant up-regulation with respect to control was shown by *P <0.05 or **P <0.01, and significant down-regulation was shown by #*P* <0.05 or ##*P* <0.01. Click here for file

Additional file 3 **The 120 abiotic stress-responsive genes identified by comparing the stress-responsive EST profiles with the previously reported abiotic stress genes from other organisms collected in Comparative Stress Genes Catalog of GCP and their expression levels in *A. mongolicus *seedlings under cold and drought conditions validated by quantitative RT-PCR.** Click here for file

Additional file 4 A list of 155 di- to hexanucleotide SSRs identified from 138 gene sequences. Click here for file

Additional file 5 **Details of primers and amplicons for qRT-PCR analysis of the 97 abiotic stress-responsive genes in *A. mongolicus*.** Click here for file
