# Supplementary material for: Identification of stress-responsive genes in Ammopiptanthus mongolicus using ESTs generated from cold- and drought-stressed seedlings
Source: BMC Plant Biol. 2013 Jun 5;13:88. doi: 10.1186/1471-2229-13-88 (PMC3679971; doi:10.1186/1471-2229-13-88)
Supplement: Additional file 2 — Table S1. Change in expression of 97 abiotic stress-responsive genes in A. mongolicus seedlings subjected to cold or drought stress. Values were color coded to represent the fold changes in expression of treatments relative to control at each time point. Significant up-regulation with respect to control was shown by *P <0.05 or **P <0.01, and significant down-regulation was shown by #P <0.05 or ##P <0.01. [file 1471-2229-13-88-S2.doc]

**Additional file 2: Table S1. Change in expression of 97 abiotic stress-responsive genes in *A. mongolicus* seedlings subjected to cold or drought stress.** Values were color coded to represent the fold changes in expression of treatments relative to control at each time point. Significant up-regulation with respect to control was shown by **P* <0.05 or ***P* <0.01, and significant down-regulation was shown by #*P* <0.05 or ##*P* <0.01.

| Contig | Accession | Annotation | *E*-value | Cold (days) | | | | Drought (days) | | | | |
| --- | --- | --- | --- | --- | --- | --- | --- | --- | --- | --- | --- | --- |
|  |  |  |  | 1 | 2 | 4 | 8 |  | 1 | 2 | 3 | 4 |
| AM1455 | Q8H8E0 | Glutathione S-transferase | 8e-64 |  | ## | ## | ## |  | ## | ## | ## | ## |
| AM0603 | O80852 | Glutathione S-transferase | 7e-80 | ## | ## | ## | ## |  | # | ## | ## | # |
| AM0617 | Q43317 | Cysteine synthase | 2e-66 |  |  |  | # |  |  |  | * | * |
| AM0025 | Q9SM65 | Catalase | 1e-145 |  |  | # | ## |  | ** | ** | * | * |
| AM1047 | Q5F305 | Beta-amylase bmy2 | 1e-92 | ** | * | ** | # |  | ** | ** | ** | ** |
| AM0598 | Q5F304 | Beta-amylase bmy1 | 1e-114 | ** | ** | ** | ** |  | ** | ** | ** | ** |
| AM0328 | Q9C598 | ATAF2 protein | 2e-45 | ** |  | ** | ** |  | ** |  | ** |  |
| AM0143 | Q9SDZ0 | Isoflavone reductase homolog 2 | 1e-104 | ** | ** | ** | ** |  | # | ## | ## | ## |
| AM1068 | Q9LMI0 | Trehalose-6-phosphate synthase 7 | 2e-58 |  |  |  |  |  | * | ** | * | ** |
| AM0509 | Q8LP14 | Nine-cis-epoxycarotenoid dioxygenase 4 | 1e-104 |  |  | ## | ## |  | ## | ## | ## | ## |
| AM0967 | Q6EJC9 | 1-deoxy-D-xylulose 5-phosphate | 1e-118 | * | * | * | # |  | * | * | ** |  |
| AM1518 | Q9SVG0 | Putative amino acid transport protein | 7e-18 | * | ** |  | ## |  | # |  |  |  |
| AM1013 | Q9AVR0 | Phosphate transporter pt1 | 1e-110 | * | * | ** |  |  | ** | ** | * | * |
| AM0878 | Q42910 | Pyruvate, phosphate dikinase | 1e-111 | ** | ** | ** | ** |  |  | # |  | ## |
| AM0970 | Q9SJM9 | Hydrolase,alpha/beta fold family protein | 1e-93 | * | ** |  | ## |  |  | * |  | # |
| AM1436 | P46519 | Desiccation protectant protein Lea14 | 1e-67 | ** | ** | ** | ** |  |  |  | ** |  |
| AM1189 | Q96453 | 14-3-3-like protein D | 1e-104 | ## | ** |  | # |  | # | ## | ## | ## |
| AM0212 | O49152 | 14-3-3 protein homolog | 1e-117 |  | * |  |  |  | # | # | ## | # |
| AM1193 | Q9M5K7 | 14-3-3-like protein | 6e-77 |  |  | * |  |  |  | # |  | # |
| AM0131 | O65357 | Aquaporin 2 Aqp2 | 2e-94 | ** | ** | ** | * |  | ## | ## | ## | ## |
| AM0828 | Q5QHW7 | Chloroplast thylakoid-bound ascorbate | 6e-63 | ** | * |  |  |  | ## | ## | ## | ## |
|  |  | peroxidase |  |  |  |  |  |  |  |  |  |  |
| AM0180 | Q76LA6 | Cytosolic ascorbate peroxidase 2 apx2 | 1e-111 | ## |  |  | ## |  | # | ## | ## | # |
| AM1069 | Q93YB2 | Putative aminoaldehyde dehydrogenase | 9e-69 |  |  |  |  |  | * | ** | * |  |
| AM0724 | Q9ZNQ4 | Superoxide dismutase[Cu-Zn] | 5e-70 |  |  | * |  |  |  | ** | ** | * |
| AM0701 | Q45QJ1 | Superoxide dismutase[Cu-Zn] | 4e-74 | ** | ** | ** | ** |  |  | ## | ## | ## |
| AM0447 | Q94IC4 | Ferritin-2 | 1e-66 | ** | ** | ** | * |  | # | ## |  | ** |
| AM0774 | Q9AR81 | Germin-like protein precursor glp3 | 8e-84 | # | ## | ## | ## |  | ## | ## | ## | ## |
| AM0466 | O24470 | Lipoxygenase | 4e-99 |  | # | ** |  |  | * | ** | * | ** |
| AM0147 | P38417 | Lipoxygenase-4 | 1e-106 | * | * | * |  |  | * | ** | ** | ** |
| AM0005 | Q9AT08 | Glucose-1-phosphate adenylyltransferase | 1e-122 | # | ** |  | ## |  |  | # | ## | ## |
| AM0463 | Q5D875 | Calcium-dependent protein kinase CDPK1 | 7e-36 |  | * | ** | ## |  | ** | ** | ** | ** |
| AM0086 | P17067 | Carbonic anhydrase | 1e-102 |  | ** | * | # |  | ## | ## | ## | ## |
| AM0981 | Q2Q064 | Carbonic anhydrase | 7e-99 | ** |  | * | ## |  | ** | * | ** | ## |
| AM0197 | Q6RIB7 | Enolase | 1e-126 | ## | ## | ## | ## |  | ## | ## | ## | ## |
| AM1353 | Q52QY1 | Ethylene response factor | 4e-45 |  | ** | ** | ** |  | ** | ** | * |  |

Table 3 (Continued)

| Contig | Accession | Annotation | *E*-value | Cold (days) | | | | Drought (days) | | | | |
| --- | --- | --- | --- | --- | --- | --- | --- | --- | --- | --- | --- | --- |
|  |  |  |  | 1 | 2 | 4 | 8 |  | 1 | 2 | 3 | 4 |
| AM1374 | Q9LKZ5 | Receptor-like protein kinase 2 | 7e-50 | *##* | ## | ## | ## |  | ## | ## | ## | ## |
| AM0667 | Q7XAE3 | Putative fructokinase 2 | 1e-120 | * | * | ** |  |  | * | ## |  |  |
| AM0630 | O65735 | Fructose-bisphosphate aldolase | 1e-96 | ** |  |  |  |  | # | # |  | ## |
| AM0903 | Q9M4M9 | Fructose-bisphosphate aldolase | 7e-61 | # | ** |  |  |  | ## | ## | ## | ## |
| AM0917 | Q6RUF6 | Fructose-bisphosphate aldolase | 1e-128 | * | * | ** | # |  | ## | ## | ## | ## |
| AM1450 | Q9LSH2 | Glutamate decarboxylase | 5e-97 | # | # | # | # |  | ## | ## | ## | ## |
| AM0595 | Q6A4W8 | Glutathione peroxidase | 1e-79 | ** | * | * |  |  | ** | ** | * | ** |
| AM0057 | P93260 | Glycolate oxidase | 1e-122 | * | ** | ** | * |  | * | ## | ## | ## |
| AM0259 | P29409 | Phosphoglycerate kinase | 1e-126 | ** |  | * | # |  | ## | ## | ## | ## |
| AM1328 | Q9LKJ2 | Phosphoglycerate kinase | 1e-87 | ## | ## | ## | ## |  | ## | ## | ## | ## |
| AM0042 | Q308Y6 | Ribulose-1,5-bisphosphate carboxylase | 1e-122 | ** | * | * | ** |  | # | ## | ## | ## |
|  |  | /oxygenase activase alpha 2 |  |  |  |  |  |  |  |  |  |  |
| AM0684 | Q40208 | RAB2A | 1e-100 | ** | ** | ** |  |  | ** | * |  | * |
| AM1171 | Q40203 | GTP-binding protein RAB1C | 5e-90 | * | ** | ** | ** |  | * | * | * | * |
| AM0790 | Q40204 | RAB1D | 2e-67 | * | ** | ** | * |  | ** | ** | ** | ** |
| AM0406 | O24550 | Malic enzyme VVME2 | 2e-81 | * | ** |  | ## |  | ** | ** |  | ## |
| AM0616 | O48923 | Cytochrome P450 | 1e-102 | * |  | ** |  |  | ** | ** | ** | ** |
| AM0845 | P17340 | Plastocyanin | 3e-58 | * | ** | ** | # |  | # | ## | ## | ## |
| AM0304 | Q8W3Y4 | S-adenosylmethionine synthetase | 1e-131 | * | ** | ** | ** |  | ## | ## | ## | ## |
| AM0346 | Q96551 | S-adenosylmethionine synthetase 1 | 1e-117 | * | * | ** |  |  |  |  | ## |  |
| AM1257 | Q45W80 | Nucleoside diphosphate kinase | 6e-77 |  |  | ## | ## |  | # | ## | # | # |
| AM0736 | Q9M4S8 | Triosephosphate isomerase | 1e-98 | ** | ** | ** |  |  | ## | ## | ## | ## |
| AM1098 | P35135 | Ubiquitin-conjugating enzyme E2 | 6e-82 |  | ** | * |  |  | * | * | ** | * |
| AM0659 | Q9SPF9 | Ubiquitin carrier protein | 3e-80 | * | ** | ** |  |  | ** | ** | ** | * |
| AM0364 | Q45W77 | Ubiquitin carrier protein | 5e-83 |  | * | ** |  |  |  | # |  |  |
| AM0976 | P35133 | Ubiquitin-conjugating enzyme E2 | 1e-80 | ** | ** | ** | ** |  | ** | ** | ** | ** |
| AM1495 | Q3HVN0 | Ubiquitin-conjugating enzyme family | 4e-57 | ** | ** | * | # |  | * | * | * |  |
|  |  | protein-like |  |  |  |  |  |  |  |  |  |  |
| AM0830 | Q42540 | Ubiquitin-conjugating enzyme E2 | 6e-44 | ** | * | ** | * |  | * | * | ** | * |
| AM0359 | Q0G879 | Peptidyl-prolyl cis-trans isomerase | 9e-37 |  | ## |  | ## |  | # | ## | # | # |
| AM0628 | Q8RVT5 | Acyl-CoA-binding protein | 4e-41 | ** | ** | ** |  |  | ** | ** | ** | ** |
| AM1165 | Q9SWA8 | Glycine-rich RNA-binding protein | 4e-35 | ** | ** | ** | ** |  | ** | ** | ** |  |
| AM0287 | P17928 | Calmodulin | 4e-79 |  |  | * | * |  | ## | ## | ## | ## |
| AM1551 | Q9SWE7 | Vacuolar ATP synthase subunit E | 1e-67 | * | ** | * |  |  | ** | ** | * | ** |
| AM1288 | P10708 | Chlorophyll a-b binding protein 7 | 1e-114 |  | # | ## | ## |  | # | ## | ## | ## |
| AM0356 | Q32904 | Light harvesting protein lhca3 | 1e-100 | # | ## | ## | ## |  | ## | ## | ## | ## |
| AM0135 | Q9SQL2 | Chlorophyll a-b binding protein P4 | 1e-123 | * | ** | ** | ## |  |  | ## | ## | ## |
| AM0017 | O48657 | Chlorophyll a/b-binding protein | 1e-105 | ** | ** | ** |  |  |  | ## | ## | ## |

Table 3 (Continued)

| Contig | Accession | Annotation | | | | *E*-value | Cold (days) | | | | Drought (days) | | | | |
| --- | --- | --- | --- | --- | --- | --- | --- | --- | --- | --- | --- | --- | --- | --- | --- |
|  |  |  |  |  |  |  | 1 | 2 | 4 | 8 |  | 1 | 2 | 3 | 4 |
| AM0022 | Q93YG3 | Chlorophyll a/b binding protein type II | | | | 1e-139 | * | ** |  |  |  | # | ## | ## | ## |
| AM0709 | Q9XQB2 | Chlorophyll a/b binding protein CP29 | | | | 1e-102 | ## | # | ## | ## |  | ** | ## | ## | ## |
| AM0084 | O81391 | Chlorophyll a/b binding protein CARCAB1 | | | | 1e-116 |  | * | * | ## |  | ## | ## | ## | ## |
| AM0106 | Q9XQB1 | Chlorophyll a/b binding protein type III | | | | 1e-126 | ** | ** | * | ## |  | ** | * |  |  |
| AM0133 | O04683 | Ferredoxin-1 | | | | 1e-38 | * | * | ** | ## |  | ## | ## | ## | ## |
| AM0665 | Q9C7Y4 | Ferredoxin | | | | 2e-58 | * | ** | ** | * |  | ** | ** | ** | * |
| AM0209 | Q6Q8B8 | Chloroplast ferredoxin I fdn-1 | | | | 5e-59 | ** | ** | ** | ## |  | # | ## | ## | ## |
| AM0262 | Q9XEN2 | Actin depolymerizing factor | | | | 1e-65 | * | ** | * |  |  | ** | ** | ** | ** |
| AM1568 | Q2PK12 | Actin depolymerizing factor-like protein | | | | 8e-68 |  | * |  | ## |  |  | ## | ## | ## |
| AM0980 | Q2HU68 | Probable histone H2A | | | | 2e-36 | * |  | ** |  |  | ** | ** | * | ** |
| AM0568 | Q6F4H4 | Actin | | | | 1e-142 |  |  |  |  |  |  |  |  |  |
| AM0925 | Q6VAF9 | Tubulin alpha-4 chain | | | | 1e-124 |  |  | * |  |  | ## | ## | ## | ## |
| AM0604 | P49679 | Auxin-induced protein IAA4 | | | | 3e-68 |  | # |  | ## |  | ## | ## | ## | ## |
| AM0618 | Q75NI2 | Type 1 metallothionein | | | | 6e-27 | ** | * | ** | * |  | * | * | ** | # |
| AM0044 | Q75NI3 | Type 2 metallothionein | | | | 3e-23 | ** | ** | ** | ** |  | ** | ** | ** | ** |
| AM1444 | P26585 | HMG1/2-like protein | | | | 3e-26 | * | * | * |  |  | ** | ** |  |  |
| AM0100 | Q9XGG5 | Fructose-1,6-bisphosphatase | | | | 4e-43 | ** | * | ## | ## |  | ## | ## | ## | ## |
| AM0898 | Q14TB1 | Heat shock protein 90 | | | | 1e-110 | ** | ** | ** | ** |  | ** | ** | ** | ** |
| AM0047 | Q9ZNQ7 | Hydrophobic protein RCI2A | | | | 3e-12 | ** | ** | ** | ** |  | ** | ** | ** | ** |
| AM0676 | Q5QHT4 | 70 kDa heat shock cognate protein 1 | | | | 1e-46 | * | * | ** | ** |  | ## | * |  | * |
| AM0734 | Q8GSN4 | Non-cell-autonomous heat shock cognate | | | | 1e-108 | * | ** | ** | ** |  | ## | ## | ## |  |
|  |  | protein 70 | | | |  |  |  |  |  |  |  |  |  |  |
| AM0696 | P09189 | Heat shock cognate 70 kDa protein | | | | 7e-61 |  |  | ## |  |  |  |  | ** |  |
| AM1245 | Q6UJ35 | Mitochondrial glycine decarboxylase | | | | 2e-73 | * | * | * |  |  | ## | ## | ## | ## |
|  |  | complex H-protein gdcH3 | | | |  |  |  |  |  |  |  |  |  |  |
| AM0772 | P16048 | Glycine cleavage system H protein | | | | 4e-74 | * | ** | ** | # |  | ## | ## | ## | ## |
| AM1536 | O81126 | RSZp22 splicing factor | | | | 3e-36 | ** | * | * |  |  | * | * | * | ** |
| AM0298 | Q6Z2M5 | Putative small nuclear ribonucleoprotein | | | | 7e-39 |  | * | ** |  |  |  | # |  |  |
|  |  |  |  |  |  |  |  |  |  |  |  |  |  |  |  |
|  |  |  |  |  |  |  |  |  |  |  |  |  |  |  |  |
| ≤0.5 | 0.51-0.99 | 1-1.99 | 2-3.99 | 4-7.99 | 8-15.99 | ≥16 |  |  |  |  |  |  |  |  |  |
